# *Gwaihir*: *Jupyter Notebook* graphical user interface for Bragg coherent diffraction imaging

**DOI:** 10.1107/S1600576722005854

**Published:** 2022-07-15

**Authors:** David Simonne, Jérôme Carnis, Clément Atlan, Corentin Chatelier, Vincent Favre-Nicolin, Maxime Dupraz, Steven J. Leake, Edoardo Zatterin, Andrea Resta, Alessandro Coati, Marie-Ingrid Richard

**Affiliations:** a Synchrotron SOLEIL, L’Orme des Merisiers, Saint-Aubin, 91192 Gif-sur-Yvette, France; b Univ. Grenoble Alpes, CEA Grenoble, 17 rue des Martyrs, 38000 Grenoble, France; c Center for Free-Electron Laser Science CFEL, Deutsches Elektronen-Synchrotron DESY, Notkestraße 85, 22607 Hamburg, Germany; d European Synchrotron Research Facility, 71 Avenue des Martyrs, 38000 Grenoble, France; SLAC National Accelerator Laboratory, Menlo Park, USA

**Keywords:** X-ray diffraction, coherence, phase retrieval, *Jupyter Notebook*, graphical user interfaces

## Abstract

Bragg coherent X-ray diffraction is a nondestructive method for probing material structure in three dimensions at the nanoscale, with unprecedented resolution in displacement and strain fields. This work presents *Gwaihir*, a user-friendly and open-source tool to process and analyze Bragg coherent X-ray diffraction data. It integrates the functionalities of the existing packages *bcdi* and *PyNX* in the same toolbox, creating a natural workflow and promoting data reproducibility. Its graphical interface, based on *Jupyter Notebook* widgets, combines an interactive approach for data analysis with a powerful environment designed to link large-scale facilities and scientists.

## Abstract 2

In a world where data are steadily made more available, *Gwaihir* is a tool that overcomes multiple issues by bridging remote access, cluster computing and a user-friendly interface, consequentially improving the link between synchrotrons and their users for Bragg coherent diffraction imaging.

## 1. Introduction

Bragg coherent diffraction imaging (BCDI) (Robinson & Harder, 2009 ▸) is a powerful technique for the nondestructive characterization of material structure in three dimensions with unparalleled spatial and strain resolution of a few nanometres (Labat *et al.*, 2015 ▸; Cherukara *et al.*, 2018*a* ▸) and 10^−4^, respectively (Newton *et al.*, 2010 ▸; Lauraux *et al.*, 2020 ▸).

The BCDI method is reliant on the coherence of the light available and thus only began to be exploited at third-generation synchrotron sources (Miao *et al.*, 1999 ▸, 2000 ▸; Robinson *et al.*, 2001 ▸). Since then it has developed into a characterization tool for *in situ*/*operando* studies of materials structure (Ulvestad *et al.*, 2016 ▸; Kim *et al.*, 2019 ▸; Carnis *et al.*, 2021*b* ▸) and will further benefit from source and beamline improvements as many synchrotrons have recently completed or are in the process of an upgrade from third- to fourth-generation synchrotrons.

BCDI relies on iterative algorithms to solve the phase lost during the measurement (Robinson & Harder, 2009 ▸). A 3D intensity distribution in the vicinity of a Bragg peak (stack of diffraction patterns forming a 3D reciprocal space map with the proper sampling) is collected from a sample illuminated with coherent light (Robinson *et al.*, 2005 ▸) and serves as input for phase retrieval. There are three main steps to complete to image the strain. The 3D map must first be pre-processed (removal of parasitic scattering intensities *etc.*; Öztürk *et al.*, 2017 ▸). It must then be inverted via phase retrieval (Miao & Sayre, 2000 ▸), the phase containing important information lost during the measurement. Finally, it is possible to extract meaningful results from the 3D complex images after post-processing (removal of phase offset, interpolation in a common orthonormal frame *etc.*).

Several software packages were developed to solve these steps but none offer a comprehensive pipeline from start to finish. For example, *PyNX* (Favre-Nicolin *et al.*, 2020*a* ▸) focuses on the phase retrieval step, *bcdi* (Carnis *et al.*, 2021*a* ▸) focuses on data pre-processing and post-processing, and *Cohere* (https://github.com/AdvancedPhotonSource/cohere) focuses on pre-processing and phase retrieval. Several graphical user interfaces (GUIs) also exist, such as *Cohere*, *Phasor* (https://github.com/DzhigaevD/phasor) and *Bonsu* (Newton *et al.*, 2012 ▸). Providing a workflow will reduce the time spent on data analysis for newcomers and improve result reproducibility by facilitating sharing while keeping track of analysis parameters and metadata.

Large-scale facilities and institutions seek ways to provide remote-access high-powered computing services to their users, which combine existing solutions in an interactive and user-friendly environment. *Jupyter* (https://jupyter.org/) is particularly advantageous and has been chosen by several institutions for such purposes, *e.g.* Google (*Colab*), the EGI federation and the European Synchrotron (*Simple Linux Utility for Resource Management*: *SLURM*).

Here we present a tool which brings together the functionality of the *PyNX* package for phase retrieval and the *bcdi* package for pre- and post-processing in a GUI built for the *Jupyter* framework (Kluyver *et al.*, 2016 ▸).

The data input/output follows NeXus definitions (Könnecke *et al.*, 2015 ▸) built in a CXI format hdf5 file (Maia, 2012 ▸). Both clarity and consistency in data formatting encourage reproducibility of the science (see https://www.panosc.eu/) and guarantee the workflow.

*Gwaihir* provides an interface to the bleeding edge of data analysis in BCDI; it can be used locally or remotely and offers an interactive and user-friendly interface with complex functionality satisfying both beginners and experts.

## 2. Software structure

**Figure 1:**
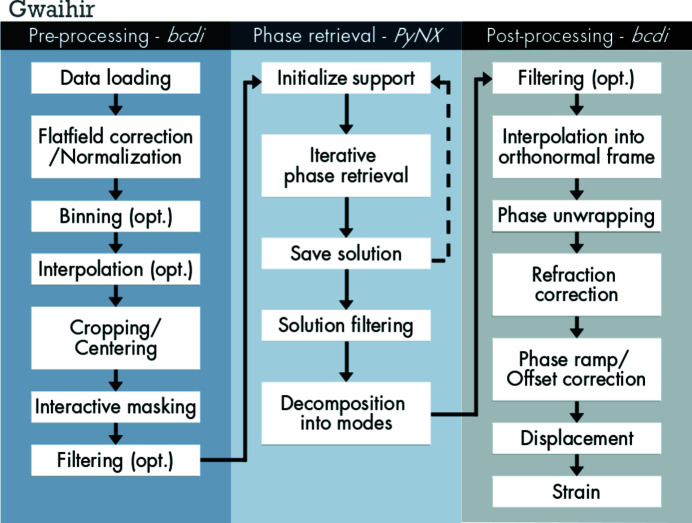
Flow chart of the main steps in the BCDI data analysis workflow. *Gwaihir* links the *bcdi* and *PyNX* packages via its GUI and command line scripts, resulting in a complete and easy to understand data analysis workflow (opt: optional).

A part of the BCDI community relies on Python, an accessible language that has gradually become one of the most popular, versatile (Perez & Granger, 2007 ▸; Newville *et al.*, 2016 ▸) and widely taught (Scopatz & Huff, 2015 ▸; McKinney, 2017 ▸; Boulle & Kieffer, 2019 ▸) programming languages in science. *Gwaihir* followed this initiative by regrouping data visualization tools, workflow guidelines and a user-friendly interface around two Python packages (*PyNX* and *bcdi*) that, together, offer a complete data analysis suite (see Figs. 1 ▸ and 2 ▸).

*Gwaihir* works with Python 3.9 and is licensed under the GNU General Public License v3.0. The package is dependent on and built around *PyNX* and *bcdi*. The source code as well as the latest developments are available on GitHub (https://github.com/DSimonne/gwaihir), while each stable version will be released on the Python Package Index (PyPi), along with its documentation.

### 2.1. Presentation of involved Python packages

#### 2.1.1. bcdi

Carnis *et al.* (2021*a* ▸) tackled the pre-processing and post-processing of BCDI data (see Fig. 1 ▸).

Data pre-processing aims to improve the quality of the phase retrieval by optimizing the size and content of the 3D array used as input. Aside from the specific details of the experimental setup (diffractometer and setup geometry, detector type, file system), the majority of the pre-processing pipeline is actually beamline independent. On the basis of this observation, *bcdi* leverages inheritance and transforms the raw data to a common data format used for phase retrieval. This frees the user from having to learn or remember the technical details for each beamline and sets a common strategy across beamlines. New *Beamline* objects can be created for coherent beamlines which are unsupported as of yet.

The minimal processing consists of loading the raw data and stacking it together as an input for the phase retrieval. Intermediate optional steps can be added via a YAML (YAML Ain’t Markup Language) configuration file. More complete pre-processing involves loading the data (with flatfield correction and masking of damaged detector pixels and detector gaps), normalizing each frame by a monitor (sensor at the beamline measuring the incoming X-ray flux), binning pixels, centering the Bragg peak and cropping the data set to a meaningful size, and interpolating missing intensities in damaged pixels. A mask is automatically created, starting from a 2D array representing the detector. It can be modified interactively to mask the parasitic scattering intensities coming from neighboring crystals via the interactive user interface. Hot pixels (saturated) or pixels with an abnormal statistical behavior are automatically masked during data loading. The result is a 3D array of the same shape as the intensity. Interpolating the data stack from the detector frame to an orthogonal frame (also called a geometric transformation) may be useful, for example, to compare Bragg peaks from different reflections. This geometric transformation can be realized using either the transformation matrix (Pfeifer, 2005 ▸) or the existing *xrayutilities* package (Kriegner *et al.*, 2013 ▸) depending on which reference basis is needed.

Post-processing regroups methods applied to the complex output of the phase retrieval. The data can be interpolated in an orthogonal frame using the transformation matrix if still in the detector frame (geometric transformation not applied during pre-processing). After phase unwrapping, a refraction correction is optionally applied, and the phase ramp and phase offset are removed. At this point the displacement and the strain component are calculated from the phase. Note that at least three non-coplanar reflections are needed to derive the complete displacement field.

#### 2.1.2. PyNX

*PyNX2011* (Favre-Nicolin *et al.*, 2011 ▸) is a toolkit with assorted Python modules and command-line scripts which can be used for the analysis of coherent X-ray imaging data, including phase retrieval (see Fig. 1 ▸). All calculations can be executed and distributed on multiple graphical processing units (GPUs) for accelerated computing using MPI.

Some facilities have started to make computational resources available to their users; those machines are an ideal environment for *PyNX* which is now available through *SLURM* at the ESRF and *GRADES* at the Optimized Light Source of Intermediate Energy of LURE (SOLEIL).

### 2.2. Graphical interface

*Gwaihir* links, in a single user-friendly and interactive GUI, the aforementioned packages while offering 2D/3D browser-based data visualization tools. The interface is built with the *ipywidgets* library (https://github.com/jupyter-widgets/ipywidgets), compatible with both *Jupyter Notebook* and *JupyterLab* web-based interactive computing platforms.

**Figure 3:**
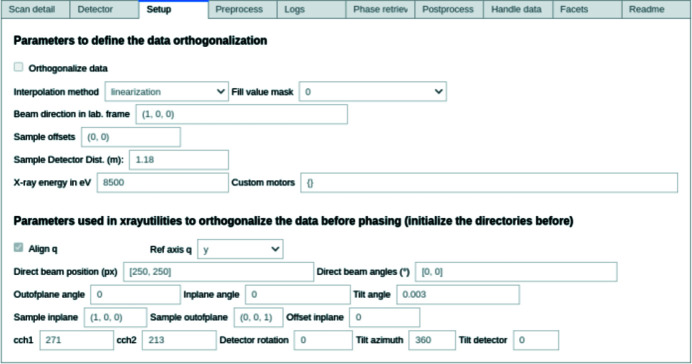
Screenshot of *Gwaihir* GUI displayed in *Jupyter Notebook*. *Jupyter Notebook* can be used on computing clusters via *JupyterHub* or on any machine for local use.

The GUI is divided into ten tabs (Fig. 3 ▸), separated into three groups: instrumental parameters, data processing and data analysis. The aim of this organization is to achieve a comprehensible but fluid workflow, while still separating each step (Section 3.1). A final tab contains information about the different methods and parameters used in the workflow, as well as a tutorial on the GUI.

#### 2.2.1. The *Jupyter Notebook* environment

The *Jupyter Notebook* environment (Perez & Granger, 2007 ▸; Kluyver *et al.*, 2016 ▸) was chosen for its versatile, user-friendly and browser-based interface.

To simplify the data analysis pipelines in fourth-generation synchrotrons, it is of critical importance to offer the possibility for external users to analyze the collected data remotely with access to computational environments

*Jupyter Notebook* has proven to be an effective tool for the analysis of synchrotron data, both in terms of the GUI (Martini *et al.*, 2020 ▸; Simonne *et al.*, 2020 ▸) and in terms of supporting scientific communities looking for high-performance frameworks (Yin *et al.*, 2017 ▸; Glick & Mache, 2018 ▸; Milligan, 2018 ▸; Stubbs *et al.*, 2020 ▸; Parkinson *et al.*, 2020 ▸). Moreover, computational environments and resources can be accessed from any computer via *JupyterHub*, a specific interface for computing clusters. *JupyterHub* offers the possibility to proceed to heavy computations without relying on specific hardware (*e.g.* GPUs) mandatory for accelerated phase retrieval with *PyNX*.

The *Notebook* can be used to take notes on the experiment or to create custom Python functions. They can be shared in .ipynb format or as .pdf documents. *Gwaihir* transforms each data set into a structured Python object that can be accessed and manipulated in the *Notebook* for *e.g.* personalized figures, parameter tests *etc.*

Moreover, researchers can create their own work spaces from shared resources, with direct access to tailored computational environments, without having to install multiple software products, keeping in mind that the use of GPUs for script optimization is far from accessible. Thus, system administrators can efficiently manage complex environments accessible to all users. Finally, remotely accessing the data avoids data storage issues, which can quickly become problematic with current CDI experiments.

#### 2.2.2. Interactive data analysis/visualization

Interactive data analysis/visualization relies on the *ipywidgets* library.

Widgets are represented in the back-end by a single object linked to a single parameter. The front-end relies on JavaScript code; each time a widget is displayed, a new representation of that same object is created. The widget style, orientation and layout attributes can be edited to customize the final window; *e.g.* the layout attribute exposes a number of properties that impact how widgets are laid out, such as height and width.

**Figure 4:**
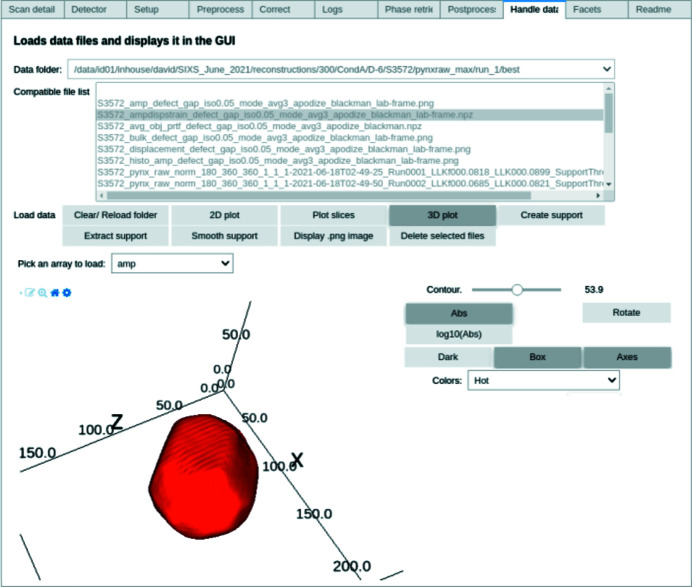
Widgets can be used to select folders and files and to tune the values of input parameters in functions. The selection of the data array, the color map and the contour of the resulting 3D object are performed through different widgets.

During manipulation of the GUI, it is possible to quickly produce a final result owing to these intuitive and interactive widgets, whose values are passed as arguments to the data analysis functions (Fig. 4 ▸). For example, to designate the type of detector used, a value must be selected in a dropdown list that comprises the following options: Eiger2M, Maxipix, Eiger4M, Merlin, Timepix. These correspond to the different detectors currently supported in the *bcdi* package.

The final interface is created by setting the different widgets on a grid, with the output of different functions printed below.

*Jupyter* natively offers multiple options for interactive data plotting. Most of the figures displayed in the GUI are based on the *matplotlib* package (Hunter, 2007 ▸). For example, it is possible to select a list of 3D complex data arrays and visualize 2D slices in each dimension of their amplitude or phase, with different color maps (diverging, sequential, cyclic) and scale (linear, logarithmic).

Moreover, an interactive 3D visualization tool is provided which relies on the *ipyvolume* library (Breddeld, 2021 ▸), itself built on top of *ipywidgets*, and is specifically designed to quickly render large 3D data arrays. A concrete application of volume rendering is shown in Fig. 4 ▸, in which a specific contour of a 3D array is represented.

In the specific case of single volume rendering for the reconstructed object, the object surface is defined by a threshold of its maximum density (Fig. 4 ▸). Finally, the object surface can be color-mapped with the values of the displacement and strain retrieved during the data analysis (see Fig. 6).

**Figure 5:**
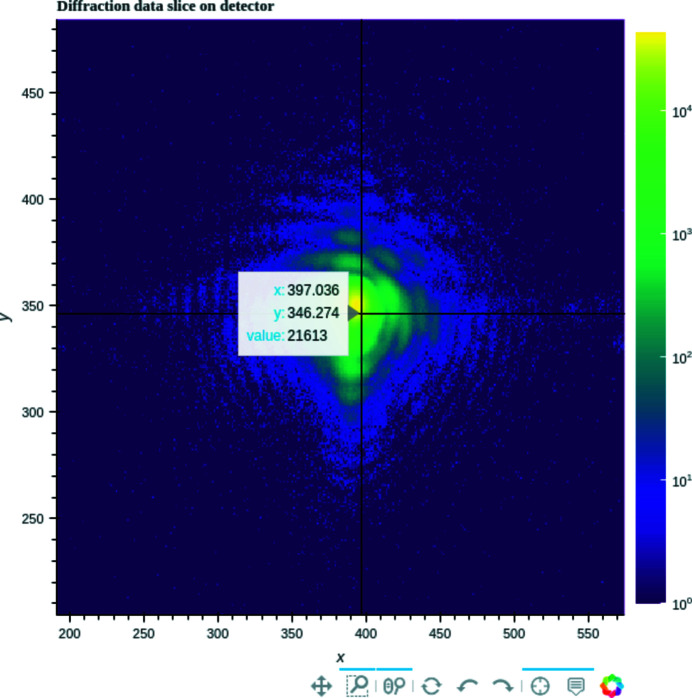
Detector images can be viewed interactively with *Bokeh*, to zoom in on the data, and visualize, for example, the intensity collected on each pixel.

To further interact with the figures (*e.g.* zoom, set the color bar range *etc.*), tools were implemented that rely on *Bokeh* (Bokeh Development Team, 2018 ▸), a Python library that transforms figures into interactive web pages (Fig. 5 ▸) that can also be displayed in *Jupyter Notebook*.

In summary, the GUI regroups data reduction, analysis and visualization tools in the *Jupyter Notebook* interface via interactive methods.

### 2.3. Command line scripts

The complete workflow for data processing can also be launched from the command line using Python scripts. In *Gwaihir*, the link between each package is based on BASH scripts. This approach is both fast and versatile, designed to quickly iterate on several data sets to test parameters values, but less intuitive.

**Figure 6:**
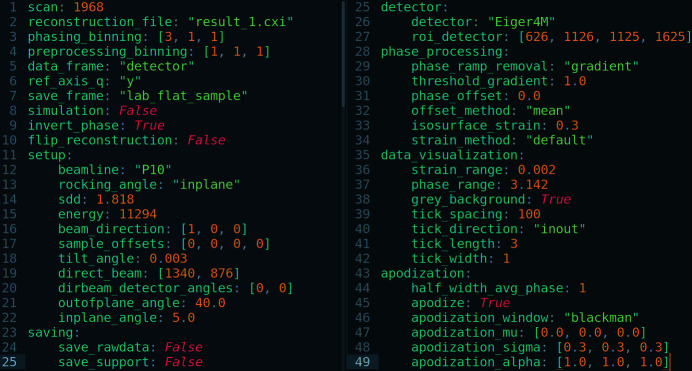
Configuration file in YAML, a human-readable data-serialization language with a minimalist syntax. Each parameter used for the analysis methods is stored. A configuration file is generated for the pre-processing and post-processing scripts, as well as for the phase retrieval.

A set of default parameters stored in a configuration file is used which can also be overwritten by providing keywords directly in the command line (*e.g.* the scan number). The configuration files are written in YAML (Fig. 6 ▸). Replication of the data analysis can be easily performed by sharing this configuration file.

## 3. Data reproducibility

Discussions about defining a set of rules that regulate research practice (Kretser *et al.*, 2019 ▸) and reduce the gray zone which includes scientific misconduct at all levels of academia (Kornfeld & Titus, 2016 ▸) are growing, raising awareness on data reproducibility in the scientific community.

Staggering numbers (Baker, 2016 ▸) show that about 65% of scientists in the field of physics and engineering struggle to reproduce others’ results, and more than 50% fail to reproduce their own. These numbers can sometimes be linked to very precise environments and techniques, with experimental conditions and processes difficult to reproduce in different laboratories, and also to knowledge transfer from academia to industry (Sarwitz, 2015 ▸). However, according to the study by Baker (2016 ▸), code availability, insufficient peer reviewing and limited access to raw data contribute to non-reproducible research.

Therefore, code availability, access to raw data combined with metadata, and reproducible workflows are goals of the utmost importance for experimental science (Munafò *et al.*, 2017 ▸). Reproducible data will result in a global improvement of confidence in new techniques, such as BCDI, which could subsequently result in growth of interest and community.

Raw data can be accessed through the CXI database (https://cxidb.org/; Maia, 2012 ▸), which aims to create a single data-storing architecture/format for coherent X-ray imaging experiments.

Data reproducibility is improved by encapsulating all the parameters and results in a final file. Therefore, any user should be able to reproduce the results from scratch. The version of each package (*Gwaihir*, *bcdi* and *PyNX*) is saved in the final file. It will be possible to use virtual environments to reproduce the same environment as that used during data analysis.

From this perspective, to improve the reproducibility of results, *Gwaihir* proposes a data analysis workflow for BCDI experiments.

### 3.1. Workflow for Bragg coherent diffraction imaging

**Figure 7:**
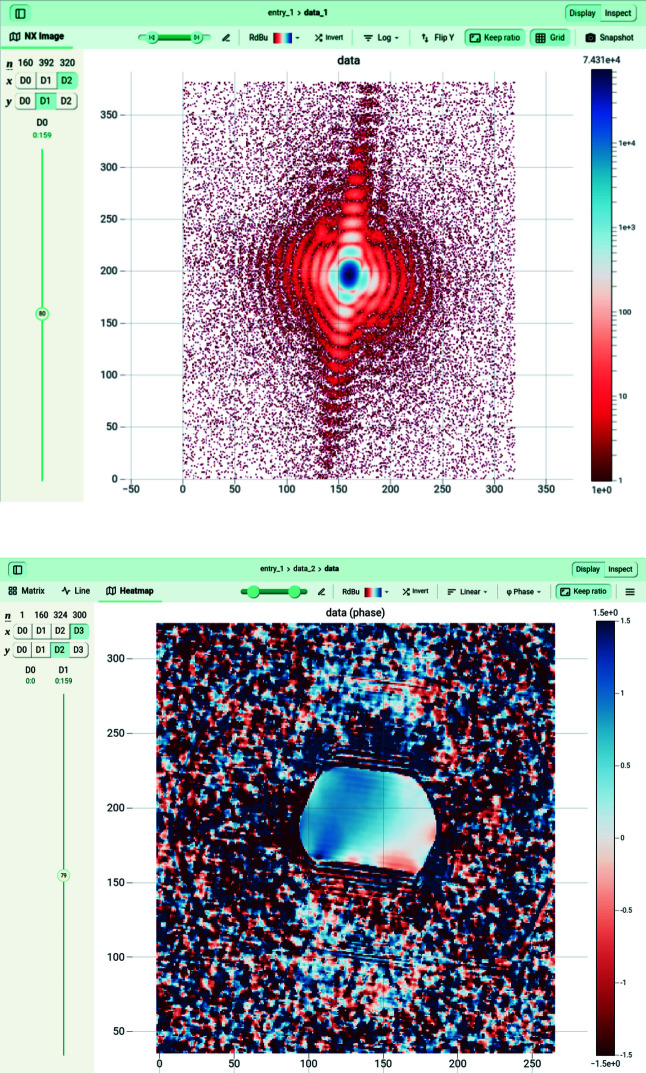
Here a 2D slice of a 3D coherent diffraction pattern is shown (top), with the phase of the electron density (radiants) of the reconstructed object in which the facets are clearly visible (bottom). The data array is displayed via *JupyterHub*, a browser-based data analysis platform available on the *SLURM* computer cluster for the ESRF and the *GRADES* computing cluster at SOLEIL.

*Gwaihir* offers an interactive workflow separated into three main groups, data pre-processing, phase retrieval and data post-processing (Fig. 1 ▸). It is intended to be reproducible and simple to share, resulting in the phase and amplitude of the probed object. To illustrate this, the results of the following procedure on a data set collected at the P10 beamline at PETRA III are shown in Fig. 7 ▸ (CXI data set ID 195).

The parameters used during the analysis are displayed in the GUI in an order following a typical workflow to underline the evolution of the data processing. These parameters are ultimately saved as attributes of the Python data object, to keep track of the actions applied to the raw data to obtain the final result.

#### 3.1.1. Pre-processing

First, the correct beamline and instrumental parameters must be selected (*e.g.* sample–detector distance, probing energy, detector pixel size *etc.*), usually constant throughout the experiment. For example, the orthogonalization tab regroups the parameters needed to properly set up the transformation matrix between the sample frame and, for example, the laboratory frame. By choosing to transform the final object in a common, orthogonal frame, it becomes easier to compare the evolution of the object when probing different Bragg reflections (Lauraux *et al.*, 2021 ▸).

Once the raw data have been collected, different pre-processing parameters can be modified to optimize the collected 3D diffraction intensity, *e.g.* the size and center of the array to analyze (either fixed manually as the center of the Bragg peak, if known, or determined as the center of mass of the 3D array during the analysis). The array can also be cropped to reduce its size, removing the points furthest from the Bragg peak where the signal-to-noise ratio is too low (Fig. 7 ▸).

It is important to create a detector mask prior to the experiment to correct the raw data for hypothetical hot pixels and background. Moreover, it is also possible to correct the raw data frame by frame for spurious data which taint the diffraction pattern. For example, it is possible, while recording the 3D diffracted intensity of a given reflection, to have signal coming either from the substrate or from neighboring objects that will be summed to the probed object intensity.

Finally, it is possible to normalize the raw data by an intensity monitor, or compute the scattering vector **q** of the measurement, from the instrumental geometry and parameters.

#### 3.1.2. Phase retrieval

The diffracted intensity *I*(*q*) collected by the detector is proportional to the squared modulus of the structure factor *F*(**q**) [equations (1) and (2)]:

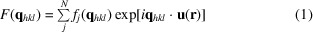

and

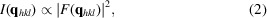

where *f_j_* (**q**_*hkl*_) is the atomic form factor of atom *j*, **q**_*hkl*_ is the scattering vector and 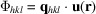 is the phase lost during the measurement; *hkl* correspond to the Miller indices of the crystallographic planes probed by the incoming beam.

The phase of the Bragg electronic density (Fig. 7 ▸) Φ_*hkl*_ is proportional to the scalar component of the displacement **u**(**r**) that is parallel to **q**_*hkl*_. A phase value of π is equivalent to a displacement from the equilibrium position of half the lattice spacing in the direction of **q**_*hkl*_.

To retrieve the phase from the diffracted intensity, *PyNX* uses iterative algorithms (see Section 2.1.2). New methods such as convolution neural networks are under study and have started to show some encouraging results, but are not yet sufficiently robust, particularly in the case of strained particles (Cherukara *et al.*, 2018*b* ▸; Chan *et al.*, 2021 ▸; Wu *et al.*, 2021 ▸).

**Figure 8:**
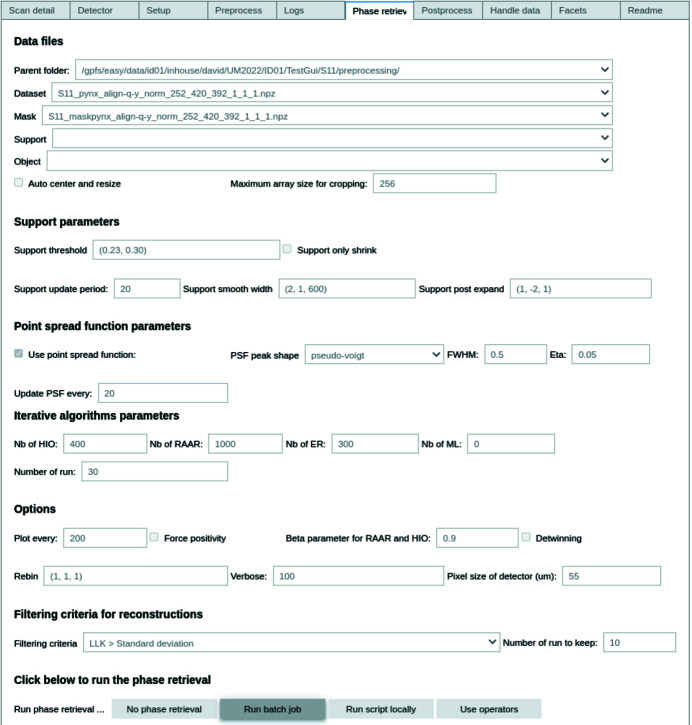
Phase retrieval tab in *Gwaihir*. Parameters are separated into groups (files, support, point-spread function, algorithms *etc.*) and detailed in the README tab. The object can be reconstructed through a batch job, submitted to the computing cluster in the back-end, or with operators that will plot the evolution of the reconstruction object in the *Notebook*.

The parameters necessary for phase retrieval can be modified in the GUI (Fig. 8 ▸), such as the support threshold, the number of iterations for each algorithm, the object initialization procedure (square, sphere, auto-correlation) and so on. Each parameter is detailed in the GUI and also discussed in the literature (Fienup, 1982 ▸, 1978 ▸; Marchesini, 2007 ▸; Favre-Nicolin *et al.*, 2020*a* ▸). Initial guesses are given for each parameter but must be refined by the user to optimize the results.

State-of-the-art GPUs available at large-scale facilities where data collection occurs enable almost real-time data reduction. This results in quick visualization of the amplitude and phase of the probed object, offering the possibility to synchrotron users to optimize their data acquisition during the experiment. Such live feedback is critical to the success of an experiment and provides broader opportunities in the type of experiments that can be carried out.

Recent updates of *PyNX*, including mathematical operators (Favre-Nicolin *et al.*, 2020*a* ▸) which represent most of the reconstruction operations traditionally used in phase retrieval (Gerchberg & Saxton, 1972 ▸; Fienup, 1978 ▸; Marchesini, 2007 ▸), laid the foundation for quick and interactive phase retrieval in *Gwaihir*.

It is possible to refine the input parameters directly in the GUI by visualizing their impact on the reconstructed object. This allows the refinement of the phase retrieval input parameters before submitting a batch job, which will spawn a sub-process on the computing cluster for phase retrieval. With a single click, several dozens of solutions can be computed in a matter of minutes on computing clusters. On a personal computer, one can use operators in the *Notebook* or spawn a job locally, yielding the same results with different execution times.

With well-tuned parameters and high-quality data sets, phase retrieval converges towards the same solution, but with minor differences between each reconstructed object, related to the phase retrieval process.

*Gwaihir* provides a wide range of selection criteria to find the best solution. Each reconstructed object has a list of final attributes that can be used as criteria for selection, such as the free log-likelihood (Favre-Nicolin *et al.*, 2020*b* ▸) or the standard deviation of the modulus of the reconstructed object. In the case of crystallographic defects, specific metrics (Chi, Sharp, Max Volume *etc.*) were derived that perform best depending on the type of object defect (Ulvestad *et al.*, 2017 ▸).

Following the selection criteria, it is possible to quickly identify the solutions of poor quality that must be excluded to create a set of best solutions. *PyNX* then offers a method that merges this set into a single solution by computing eigen-vectors for the selected solutions (Favre-Nicolin *et al.*, 2020*b* ▸). An alternative approach also available in *Gwaihir* is to take the average of the best solutions (see *e.g.* Ulvestad *et al.*, 2014 ▸).

#### 3.1.3. Post-processing

Once the solution with the best figure of merit is selected, it is possible to use *bcdi* scripts to process the phase of the object. The phase origin can first have an impact when comparing the lattice displacement between different reconstructions. In the case of weak strain, it can be sufficient to consider the center of mass of the object as the origin of the phase. However, this can become quite complex in the case of defects or defaults in the object. Special methods are defined to target this issue in *bcdi*. For example, Guizar-Sicairos *et al.* (2011 ▸) and Hofmann *et al.* (2020 ▸) proposed a convenient method for the numerical calculation of phase gradients in the presence of phase jumps. More details and guidelines are given by Carnis *et al.* (2019 ▸, 2021*a* ▸) as well as in the README tab of the GUI. Different methods can be tested and their results directly compared via the visualization tab.

#### 3.1.4. Facet analysis

The surface in BCDI corresponds to the surface voxel layer defined by a threshold of its maximum density. Guidelines on how to select this threshold are given by Carnis *et al.* (2019 ▸), the objective being to correctly select the surface voxels of the object.

**Figure 9:**
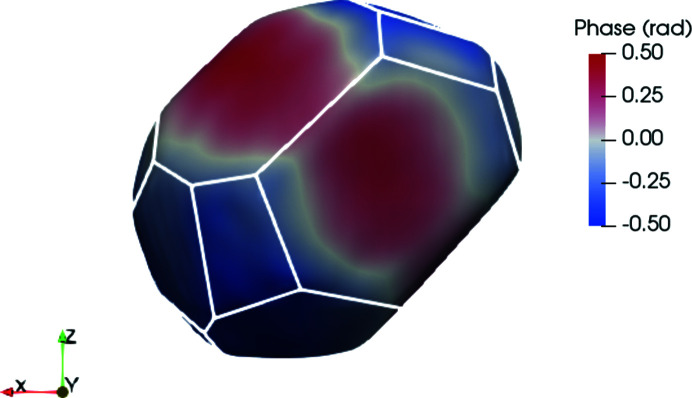
In this screenshot taken from *Paraview*, the particle facets are identified by the white contours. The coloring represents the phase of the reconstructed object, proportional to the lattice displacement. This particle has a diameter of about 800 nm.

Once the threshold for the iso-surface is selected, it is possible to visualize a contour of the object directly in the GUI (Fig. 4 ▸) with specialized solutions such as *Paraview* (Ahrens *et al.*, 2005 ▸) (Fig. 9 ▸). Crystallographic facets can be identified on the surface of the reconstructed object when studying faceted objects with a highly coherent beam (Richard *et al.*, 2018 ▸), allowing in-depth studies of facet-dependent strain and displacement. Lattice strain and displacement are key factors in fields such as heterogeneous catalysis (Ulvestad *et al.*, 2016 ▸; Kim *et al.*, 2018 ▸; Fernández *et al.*, 2019 ▸; Passos *et al.*, 2020 ▸; Carnis *et al.*, 2021*b* ▸) or electrochemistry (Vicente *et al.*, 2021 ▸).

Retrieving the facets can be achieved by analyzing the probability distributions of the orientations of triangle normals on a mesh representation of the object (Grothausmann *et al.*, 2012 ▸). This method is used in the *Paraview* plugin *FacetAnalyser* (Grothausmann & Beare, 2015 ▸), and yields a list of features detailed in Table 1 ▸. A similar method is used in *bcdi*.

In this example, we use the *FacetAnalyser* plugin (Fig. 9 ▸). Note that the edges and corners, sites of particular interest for heterogeneous catalysis (Taylor & Armstrong, 1925 ▸), are also retrieved together as voxels not belonging to any facets. The result depends on the algorithm input parameters, such as the minimum relative facet size, the angular acceptance for the facet normals *etc.*

The .vtk output file of *FacetAnalyser* can be opened and manipulated in the GUI. Each facet is assigned a crystallographic orientation that allows *in fine* facet-dependent structural analysis. The facets can be visualized individually in three dimensions in the GUI, together with the edges and corners, to help understand the particle shape.

### 3.2. Resolution

Finally, the spatial resolution of the reconstructed object, defined according to multiple parameters in the literature, is critical for imaging techniques.

The voxel size in real space depends on the collected volume of reciprocal space: 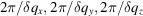 , where *q_x_*, *q_y_*, *q_z_* are the coordinates of the scattering vector. Three methods are commonly used to estimate the spatial resolution.

First, the phase retrieval transfer function (PRTF) (Chapman *et al.*, 2006 ▸) is the ratio of the calculated amplitude to the measured amplitude as a function of the resolution ring, which is a fraction of the sampling frequency of the diffraction pattern. The relative frequency at which the PRTF is equal to 0.5, or to 1/*e*, can be used as an estimated spatial resolution of the reconstruction. Note that Cherukara *et al.* (2018*a* ▸) have recently demonstrated that the resolution of 3D real-space images obtained from Bragg X-ray coherent diffraction measurements is direction dependent.

Secondly, the Fourier shell correlation (van Heel & Schatz, 2005 ▸) measures the normalized cross-correlation coefficient between two 3D volumes and hence depends on two reconstructions that have to be the result of independent data sets.

Thirdly, spatial resolution can be quantified by differentiating line profiles of electron density amplitude across the object–air interface and fitting these with a Gaussian profile. The average 3D spatial resolution is taken as 2σ of the fitted Gaussian (Hofmann *et al.*, 2020 ▸).

Concerning the resolution of the retrieved atomic displacement, Labat *et al.* (2015 ▸) have demonstrated a displacement field accuracy of a few picometres with BCDI.

The resolution can be quantified in the *Gwaihir* GUI following different criteria and shared along with the processed data. Testing the influence of different parameters on the resolution and final results will help to derive optimum parameter values for data analysis.

### 3.3. Data storage

Since not all beamlines provide self-explaining NeXus data sets, it is the *bcdi* package that allows the support of the coherent imaging beamlines ID01 (ESRF), P10 (PETRA III), SixS and CRISTAL (SOLEIL), NanoMAX (MAX IV), and 34-ID-C (APS). Data pre-processing will generate two files stored as *NumPy* arrays (Van der Walt *et al.*, 2011 ▸), corresponding to the diffraction intensity and mask. These two files are then used for phase retrieval, for which the final object is saved in a CXI file, later used for data post-processing. In the case of simulation, the simulated diffraction intensity can be stored as a *NumPy* array to start the workflow from phase retrieval.

**Figure 10:**
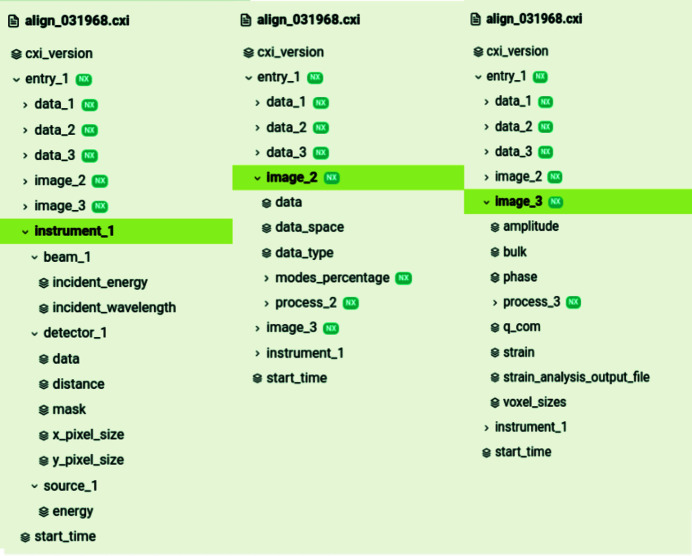
Each parameter used during the data analysis is stored in the same .cxi file, along with the results, in a nested architecture: ‘instrument_1’ regroups parameters associated with the instrumental setup; ‘data_1’ is the diffraction pattern collected; ‘image_2’ regroups the parameters associated with phase retrieval; ‘data_2’ is the reconstructed Bragg electronic density chosen for post-processing; ‘image_3’ regroups parameters linked to data processing, as well as the processed amplitude, phase and resulting strain; ‘data_3’ is a link to the processed phase of the object. The file structure is displayed via *JupyterHub*.

In *Gwaihir*, data sharing across teams and team members is facilitated by the creation of a single output file, respecting the CXI (Maia, 2012 ▸) and thus the NeXus (Könnecke *et al.*, 2015 ▸) architectures. As written by Könnecke *et al.* (2015 ▸), authors of data reduction and data analysis software can use NeXus to store processed data along with metadata and a processing log. This is illustrated in Fig. 10 ▸, in which the CXI data set tree is displayed. It regroups parameters from phase retrieval together with parameters from pre-processing and post-processing.

The raw data, along with all the parameters associated with the analysis, and the final results are stored in a single CXI file, allowing complete data analysis reproducibility (Fig. 10 ▸). This is possible at every step of the workflow. The aim is first to have a comprehensible architecture and secondly to be able to reproduce others’ results from this file. On a small scale, results are easier to share between collaborators and are more understandable, whereas on a larger scale it will facilitate peer review.

Key parameters for data reproducibility are the transformation matrix used for the final interpolation, the voxel size of the resulting 3D array, the probed reciprocal space range after data pre-processing (δ*q_x_*, δ*q_y_*, δ*q_z_*), the iso-surface threshold *etc*. By sharing these parameters along with the diffraction intensity and complex Bragg electronic density, we can gain more transparency in the methods used to obtain the final results. Comments or metadata, such as the horizontal and vertical coherence lengths, or beam size, if determined prior to the experiment, can also be saved.

### 3.4. Examples

Example data can be downloaded from the CXI data bank (Maia, 2012 ▸). We have shown in Fig. 7 ▸ an example of an output file, using data collected at P10 (PETRA III; CXI entry 195). The GUI was tested on data collected at the ID01 beamline (ESRF; CXI entry 182) and also at the SixS beamline (SOLEIL; CXI entry 194), for which it is used as an ‘online’ analysis tool.

Demonstration videos detailing the GUI can be found in the GitHub repository at https://github.com/DSimonne/gwaihir. The .cxi file that resumes the data analysis workflow can be downloaded at https://www.dsimonne.eu/PhDAttachments/align_031968.cxi.

## 4. Conclusions

*Gwaihir* is a versatile tool for BCDI data analysis. It puts state-of-the-art BCDI analysis tools at researchers’ fingertips in an intuitive interface and exploits the *Jupyter* framework to provide either local or remote operation seamlessly.

*Gwaihir* can interrogate the data in two or three dimensions to exploit the computing power of cluster resources. It offers the possibility to share data in CXI format across users, a common architecture that includes all parameters and results of the methods used during pre-processing, post-processing and phase retrieval, and enables consistent and reproducible data analysis.

In a world where data are steadily made more available, *Gwaihir* is a tool that overcomes multiple issues by bridging remote access, cluster computing and a user-friendly interface, consequentially improving the link between synchrotrons and their users. 

## Figures and Tables

**Figure 2:**
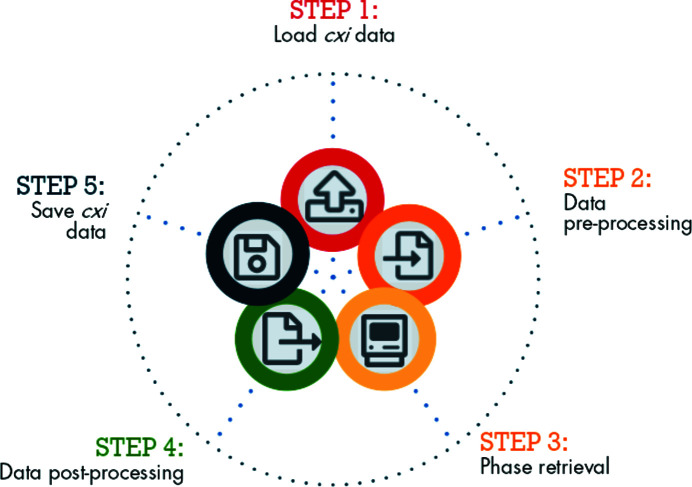
Workflow steps taken in *Gwaihir*; the circular workflow illustrates data reproducibility, a key concept, facilitated by the CXI architecture. The user may start the analysis directly from phase retrieval or post-processing, after reloading the CXI file.

**Table 1:** The output of facet analysis is a list of values for each facet The accessible features are facet size, average strain, average displacement, the facet center and the facet normal. The uncertainty on the average displacement and strain corresponds to the standard deviation of the displacement and strain distribution, respectively.

Facet ID	Facet normal	Relative facet size	Average displacement (Å)	Average strain (10^−4^)
1	(1, 1, 1)	0.106	0.080 ± 0.173	−0.28 ± 0.51
2	(1, 1, 1)	0.223	0.052 ± 0.26	−1.17 ± 0.64
3	(1, 1, 0)	0.106	0.080 ± 0.173	−0.28 ± 0.51
4	(1, 0, 0)	0.096	0.137 ± 0.192	0.27 ± 0.49
0	Edges and corner	NaN	−0.224 ± 0.259	0.20 ± 0.86
